# Impact of Community Midwifery Care (CMC) on maternal and neonatal wellbeing in postnatal period: a qualitative study

**DOI:** 10.1186/s12884-026-08879-8

**Published:** 2026-03-13

**Authors:** Cristina Lumia, Matilde Flossi, Antonella Nespoli

**Affiliations:** 1 School of Medicine and Surgery, University of Milano-Bicocca, Via Cadore n. 48, Monza, 20900 Italy; 2 School of Medicine and Surgery, Insubria University of Varese, Varese, Italy

**Keywords:** Qualitative research, Midwifery, Postpartum period, Mothers, Neonatal nursing

## Abstract

**Background:**

Puerperium is an extremely delicate and sensitive period in a woman’s life, during which she needs to be supported by family and healthcare professionals. The midwife’s role is supporting the mothers wishes and address her concerns. The aim of this study is to investigate the impact of Community Midwifery Care (CMC) on maternal and neonatal wellbeing through the description of women’s experience.

**Methods:**

Data were collected through semi-structured interviews in a qualitative study from April to August 2024. No exclusion criteria were applied other than having received CMC. Women were included regardless of the type of labor or birth (spontaneous, induced, augmented, or surgical) but all of them had given birth in one Italian hospital. One researcher conducted all interviews and women recruitment continued until thematic saturation was reached (*N* = 24). Analysis was performed with NVivo Software.

**Findings:**

Mother’s age ranged from 19 to 41 years old. Two main themes and five sub-themes were extracted. Main themes included concerns related to the puerperium and knowledge of CMC services. Neonatal care, breastfeeding, maternal care, resumption of sexual activity after childbirth and participation to pre- and post-partum classes were found as sub-themes.

**Conclusions:**

Community midwifery care improved mother’s wellbeing by providing personalized emotional support, increasing breastfeeding confidence, and facilitating timely management of postnatal apprehensions. Strengthening continuity of care and integrating CMC into standard postnatal practices might improve women’s initial postnatal experiences and improve newborn outcomes.

## Background

The postnatal period is recognized as a vulnerable transition involving physical recovery, emotional adaptation and the acquisition of new parenting skills. Adequate postnatal care is therefore essential to promote maternal wellbeing, breastfeeding success and healthy infant growth [1]. Midwives play a central role in this phase by providing clinical assistance, counselling and continuity of care during early motherhood [2, 3].

Evidence highlights that postnatal midwifery surveillance may reduce anxiety, support breastfeeding and facilitate early problem-solving in newborn care [4, 5]. In global policy, the State of the World’s Midwifery Report emphasizes the relevance of community-based models of care in postpartum support [6], although evidence specific to the postnatal period remains limited compared to antenatal and intrapartum settings. Research supporting midwife-led continuity of care is extensive [7], yet postpartum outcomes such as maternal mental health, pain, breastfeeding challenges, sexuality and parental transition remain underexplored.

Community Midwifery Care (CMC) provides continuity after hospital discharge through home visits and counselling, offering a safe space for questions, reassurance and timely support. Research shows that women value personalized follow-up, describing community care as relieving, empowering and confidence-building [8].

In Italy, national attention to postnatal support has increased and the Family and Community Midwife role has been formally proposed (*Legislative Decree 2076/2021).* In our setting, CMC is provided through home visits and/or community health center facilities (*Consultorio*, in Italian language and context). First contact generally occurs within 7–10 days postpartum, with frequency adjusted to individual needs. Support may continue up to 3–6 months, including: breastfeeding counselling; neonatal care guidance (bathing, cord care); maternal physical recovery monitoring; emotional support and early detection of psychological distress; contraceptive and sexual counselling referral to multidisciplinary team if necessary [9]. This flexible, woman-centred approach allows midwives to intervene during key transition moments, facilitating maternal confidence, self-efficacy and access to resources.

Despite international recognition of postpartum midwifery care, little is known about women lived experiences of community-based support and which aspects they value most. Understanding these perspectives is crucial to improve postnatal pathways and guide service planning. This qualitative study explores women’s experiences of Community Midwifery Care after childbirth, with a focus on how midwives support shapes confidence, caregiving, emotional recovery and engagement with local services. Findings aim to inform woman/family-centred models and strengthen community-based postnatal care.

## Methods

### Study design

We conducted a qualitative descriptive study [10], selected to provide a straightforward and pragmatic description of women’s experiences with community midwifery care in the postnatal period. This approach allows data to remain close to the participants’ words and meanings, without imposing theoretical interpretation [11].

### Research questions

1. How do women describe their experience of Community Midwifery Care in the postnatal period?
2. Which components of CMC are perceived as supportive for maternal and neonatal wellbeing?
3. What needs, challenges or suggestions for improvement emerge from women’s perspectives?

### Setting

The study was conducted in a public hospital in Northern Italy, where Community Midwifery Care (CMC) is offered in the postnatal period. The service includes home-based postnatal visits, carried out by community midwives to support maternal and neonatal wellbeing through clinical assessment, breastfeeding support and counselling.

### Participant recruitment and sampling

Eligible participants were mothers who had received CMC within the previous 3–6 months of any nationality, with diversification in age and socioeconomic background, who had given birth at Varese Hospital and were hospitalized in the Obstetrics ward. Midwives (CL and MF) acted as gatekeepers and introduced the study during routine clinical interactions. Women who expressed initial interest gave verbal permission to share their contact details with the research team. An initial list of eligible women was therefore drawn up. This included their telephone and email contact details. A researcher (MF) subsequently contacted them by phone, provided detailed study information and obtained written informed consent prior to scheduling the interview. Women who were not available for interviews after signing the consent form were excluded from the study.

We adopted purposive sampling to obtain variation in age, parity, breastfeeding experiences and type of midwifery support received. No exclusion criteria were applied other than not receiving CMC, and mothers were included regardless of mode of birth (spontaneous, induced, augmented or caesarean section). Recruitment continued until data saturation was reached, defined as no new relevant codes emerging in at least two consecutive interviews [12].

A total of 24 women were included in the final sample. Data saturation was achieved within the final interviews, with no new relevant codes emerging in the last two interviews. This sample size is consistent with methodological guidance for qualitative interview studies in health research, where sample adequacy is determined by information power rather than numerical representativeness [13].

### Data collection

Data were collected from April to August 2024 through face-to-face semi-structured interviews, conducted by the first author (CL) trained in qualitative methods. Each interview lasted 30–45 min, was audio recorded with consent and transcribed verbatim. Field notes were written immediately after each session.

### Interview guide

The interview guide was informed by literature on postnatal care and reviewed by a senior midwifery researcher (AN) to ensure clarity and relevance. A pilot interview tested flow and language.

Key explored topics included:

- experience of postnatal CMC;
- emotional, informational and practical support received breastfeeding and infant care;
- perceived benefits and challenges;
- suggestions for improving CMC.

Example questions: *“Can you describe how the midwife supported you after birth?”*,* “What aspects of care helped you the most/least?”*,* “How did this support influence your confidence as a mother?”*

(see Table 1 for more details). Five pilot interviews were conducted but these were not included in the research findings.

**Table 1:** Examples of interview guide questions

Domain explored	Example prompts used during interviews	Linked aspects/outcomes
Maternal experience with CMC	“*Can you tell me about your postnatal experience and how the midwife supported you?”*	Perceived usefulness, continuity of care, confidence
Breastfeeding & infant feeding support	“*How did the midwife assist you with breastfeeding or bottle-feeding? What helped or what was missing?*”	Establishment of feeding, difficulties, reassurance
Daily newborn care & routines	“*How did you manage newborn care at home? Did the midwife help you feel more confident?”*	Baby care skills, self-efficacy, autonomy
Emotional wellbeing & maternal adjustments	“*How did you feel physically and emotionally in the early weeks? Did CMC influence your wellbeing?”*	Anxiety reduction, reassurance, sense of safety
Relationship & communication with midwives	*“How would you describe your interaction/relationship with the midwife?”*	Trust, bonding, continuity, accessibility
Perceived benefits and challenges of CMC	*“What aspects were most useful or less helpful? Any barriers?”*	Strengths/limitations of the model
Suggestions for service improvement	*“What could improve the service for future mothers?”*	Practical recommendations, unmet needs

### Data analysis

Data were analyzed using reflexive thematic analysis following Braun & Clarke’s [14] six steps: (1) familiarization, (2) initial coding, (3) theme generation, (4) theme review, (5) definition and naming, and (6) synthesis.

All data were managed using NVivo 15. A reflexive approach was maintained through analytic memos acknowledging the researcher’s professional background as a midwife. Themes were refined iteratively through peer debriefing with the supervisory team.

### Trustworthiness and reflexivity

Methodological rigor was enhanced following COREQ principles [15]. An audit trail was maintained throughout the study, including coding memos and iterative analytic decisions. Two researchers (CL and MF) independently coded transcripts and compared interpretations, and disagreements were resolved through discussion, improving dependability. Credibility was supported through verbatim quotations used to main themes in participants’ narratives. Data adequacy was supported by the achievement of saturation, as no new relevant insights emerged in the final interviews. Peer-debriefing sessions within the research team were conducted during analysis to challenge assumptions and refine theme development.

Reflexivity was addressed by the primary researcher (CL) keeping reflective notes to acknowledge her professional background as a midwife and potential influence on data interpretation. Regular discussions within the team helped monitor positionality, bracket preconceptions, and ensure interpretations remained women-centered.

### Ethics

This study was conducted in accordance with the ethical principles of the Declaration of Helsinki. Ethical approval was obtained from the Research Ethics Committee of Insubria University of Varese (Italy). Written informed consent was obtained from all participants prior to data collection. All data were anonymized and handled in accordance with GDPR regulations.

Participants were informed that the interviews would be stored for research reasons and then published in a peer-reviewed journal in an anonymous format. The sampled women were assigned numerical identifiers (W1, W2, W3, …).

### Findings

Twenty-four mothers were interviewed and four withdrew before the interview stage. Participants were aged 18–41 years (mean age 31.6). Most were Italian, with two non-Italian women (Ukrainian and Serbian). Educational level ranged from high school to master’s degree, and family status varied across married, cohabiting and single mothers (see Table 2).

**Table 2:** Characteristic of the participants (*N* = 24)

Woman ID	Age	Nationality	Personal status	Education
W1	19	Italy	Married	Diploma
W2	30	Italy	Not married	Diploma
W3	37	Serbia	Married	Bachelor’s Degree
W4	33	Italy	Married	Diploma
W5	40	Italy	Not married	Diploma
W6	22	Italy	Not married	Diploma
W7	35	Italy	Not married	Bachelor’s Degree
W8	39	Italy	Married	Master’s Degree
W9	33	Italy	Single	Diploma
W10	40	Italy	Married	Master’s Degree
W11	25	Italy	Married	Diploma
W12	39	Italy	Married	Bachelor’s Degree
W13	24	Italy	Not married	Diploma
W14	36	Italy	Married	Diploma
W15	20	Italy	Not married	Diploma
W16	34	Italy	Married	Master’s Degree
W17	39	Italy	Married	Master’s Degree
W18	18	Italy	Single	Diploma
W19	41	Italy	Married	Bachelor’s Degree
W20	32	Italy	Not married	Diploma
W21	37	Ukraine	Married	Diploma
W22	26	Italy	Married	Diploma
W23	21	Italy	Not married	Diploma
W24	40	Italy	Married	Bachelor’s Degree

Analysis generated two overarching themes and five sub-themes (see Table 3). Below, themes are presented with interpretative commentary supported by illustrative quotes in line with qualitative reporting standards.

**Table 3:** Themes and sub-themes extracted from the data analysis

Themes	Sub-themes
Dealing with the Puerperium: necessities, uncertainties, and physiological modifications	Neonatal care as the first stage of learning
	Breastfeeding: support, reassurance and informed choice
	Maternal well-being and self-care often come last
	Resuming sexual life: silence, fear and confidence
Knowing (or not knowing) the service: discovering what midwives can offer	Postnatal classes as door to empowerment

### Theme 1. Dealing with the puerperium: necessities, uncertainties, and physiological modifications

Women described the postpartum period as a fragile transition marked by physical recovery, emotional fluctuation and the need to integrate the newborn into family life. Many expressed uncertainties about “*doing things right*” and a desire for professional reassurance, particularly in the first weeks at home.

*“When I came home*,* I felt I should already know everything… but I didn’t. Having someone tell me step by step made me breathe.”* (W20)

Midwifery support was repeatedly described as a source of confidence, continuity and calmness, enabling mothers to feel less alone in decision making.

*“When I got home*,* everything felt new… I was happy but overwhelmed. The midwife helped me understand that what I was feeling was normal.”* (W1)

Although several women already had older children, many described the second experience as emotionally different and sometimes more challenging, reflecting that experience does not necessarily remove vulnerability.

*“Even though it was my second baby*,* I needed someone to refresh things — everything changes and I didn’t want to do things wrong.” (W17)*

Community midwifery care emerged as a protection in the adjustment process, helping women regain balance and normality.

### Sub-theme 1.1 - Neonatal care as the first learning curve

Neonatal care was often narrated as a moment of fear mixed with discovery, particularly for first-time mothers. Practical guidance on bathing, cord care and handling the baby helped transform fear into familiarity.

*“He looked so tiny*,* I was afraid to dress him wrong… the midwife showed me slowly*,* and it felt possible.”* (W22)

In addition, the changes in care that we see every day can be managed with the timely and professional support of a midwife.

*“My first is six years old; I had forgotten everything… the midwife reassured me that things have changed since then. Besides*,* I was able to use hydrogen peroxide with him for cord care*,* and now the midwife told me I don’t need to put anything on”* (W3)

Women associated hands-on support with empowerment, highlighting that even small demonstrations reduced anxiety and facilitated bonding.

### Sub-theme 1.2 - Breastfeeding: support, reassurance and informed choice

Many women started with the intention to breastfeed., but feeding was described as physically demanding and emotionally charged.

“*Breastfeeding was harder than I imagined… I cried the first nights*,* but then the midwife came and things felt lighter.” (W15)*

Access to timely midwifery advice was identified as a turning point both for continuing breastfeeding and for feeling legitimized when choosing to stop.

*I called the health community centre (*Consultorio, in Italy*) straight away to ask my midwife how to deal with what I thought was mastitis*,* but in the end*,* she told me it was just a temporary engorgement*,* and I went for a check-up the next day*”. (W4)

Women’s experiences highlighted how midwives acted as trusted figures who validated emotions and guided choices without judgement. One participant shared:

“*I decided to stop breastfeeding just before my discharge from hospital and I felt judged*,* luckily the community midwife explained to me how to prepare and store milk*,* otherwise I would have looked on the internet*”. (W19)

Breastfeeding was therefore not only a technical practice, but a relational experience shaped by reassurance rather than pressure.

### Sub-theme 1.3 - Maternal well-being and self-care often come last

Women reported that their own needs were frequently overshadowed by the newborn’s, with self-care perceived as optional unless prompted by a professional.

*“Everyone asked how the baby was… no one asked how I was.”* (W11)

Many expressed difficulties in finding time for rest, wound care and emotional decompression.

*“I had read on the internet that young mothers are more likely to get sick*,* so the feeling of inadequacy was already feeling like depression. My midwife told me about a week after the birth that what I was feeling was normal*,* that I would get better in time and that we all feel inadequate at the beginning”. CMC can arrange to see a psychologist if necessary.* W1 continued: *“I had never used psychologists before*,* but coming from a midwife I trusted*,* it seemed like a good idea”*

Community midwives were valued for normalizing emotions, checking on sutures or wound healing, and signposting psychological support when needed. Care for the mother was recognized not as secondary but as integral to newborn well-being.

### Sub-theme 1.4 - Resuming sexual life: silence, fear and confidence

Sexuality was often described as delayed, painful or obscured by tiredness, yet rarely discussed spontaneously.

*“I didn’t feel ready*,* not physically but emotionally… I needed someone to tell me it was okay to take my time.” (W21)*

Where addressed, midwives offered reassurance and body-healing information that relieved fears of pain or injury recurrence.

*I was always tired; I was afraid that the stitches would not heal properly. In the end*,* when I went to the Consultorio (CMC)*,* the midwives reassured me that after the initial fear everything would be fine and that the stitches had already fallen out”*

Sexual adjustment appeared to be a slow and highly individual process, with women valuing open discussion free from judgement.

### Theme 2. Knowing (or not knowing) the service: discovering what midwives can offer

Awareness of community midwifery was fragmented and uneven. Many women learned about services only after birth, often by chance or through a professional. Lack of information limited access, whereas supportive encounters opened the door to empowerment beyond clinical care.

*“I didn’t know midwives did all this… once I met them*,* I realized how much support there is.”* (W7)

### Sub-theme 2.1 - Postnatal classes as door to empowerment

Postnatal groups were perceived as useful spaces for learning and connection, although not all women attended or knew they existed. Those who participated valued discussions on breastfeeding, psycho-physical changes and pelvic floor rehabilitation.

*“I wish someone had told me earlier… I looked online and got even more confused.”* (W24)

For several women, sharing emotions and concerns within a supportive space contributed to normalizing their feelings and reducing the sense of inadequacy often experienced in early postpartum. This was captured by W20:

*“Talking to other mothers and midwives made me feel normal again.”* (W20)

Women suggested strengthening communication about available services and expanding classes to include pelvic floor care since pregnancy, emotional wellbeing and practical newborn topics.

## Discussion

This study explored women’s experiences of Community Midwifery Care (CMC) during the early postnatal period, illuminating how midwife-led support shaped maternal confidence, breastfeeding experience, emotional wellbeing and provision of newborn care. Collectively, the findings suggest that CMC worked not only as clinical assistance, but as an *emotional anchor* fostering reassurance, normalization and safety during the transition to motherhood.

These findings are consistent with international evidence on postnatal home visiting and community-based midwifery programmes, which also similarly identify relational continuity, individualized support and education as central mechanisms facilitating women’s transition to motherhood [16]. A qualitative systematic review of postnatal midwifery home care literature has reported that women–midwife connection, recognition of individual needs and supportive education were key elements underpinning maternal confidence and adjustment in early motherhood [17].

Similarly, qualitative studies of midwifery home visits have shown that relational continuity and the opportunity to discuss experiences in the home environment enhance women’s sense of safety, confidence and emotional recovery in the early postnatal period [18]. According to previous research, the immediate postpartum phase emerged as a delicate and transformative period marked by physical recovery, fatigue, unfamiliar bodily sensations and uncertainty in caring for the newborn [19, 20]. Many mothers described feeling emotionally unstable and insecure about their abilities as parents, which mirrors global literature reporting that early postpartum requires intensive relational support to mitigate anxiety and foster maternal identity development [21, 22]. Our findings show that midwives played a central role in strengthening maternal self-efficacy [23, 24]: through calm presence, gradual teaching and validation of emotions. Practical support such as assistance with breastfeeding challenges, bathing, cord care or wound management helped transform fear into familiarity and promoted bonding. Evidence indicates that relational reassurance and supportive interactions [21] can reduce psychological distress and lower the risk of postpartum depression [25, 26].

These observations align with our earlier work exploring women’s postnatal experiences, where continuity of midwifery support similarly enhanced emotional safety and trust during early motherhood [9]. Women described midwives as stabilizing figures who reduced uncertainty through presence rather than instruction alone - highlighting how care *is felt*, not only *delivered*.

Breastfeeding was narrated as both desired and demanding, echoing the wider literature describing it as a site of emotional pressure, body negotiation and social expectation [27, 28]. Women appreciated timely, non-judgmental guidance that helped them continue breastfeeding when difficulties arose, but equally valued being supported when choosing to stop [29]. This confirms evidence that mother-centered counselling improves wellbeing and reduces guilt associated with infant feeding decisions [30, 31]. Community midwives enabled informed choice, offering advice on preparation and storage of formula when breastfeeding was not possible or desired.

Postnatal sexuality appeared as a silent, often overlooked dimension of recovery. Women reported limited prioritization initially but lower satisfaction months later: a finding consistent with studies showing that sexual wellbeing frequently declines postpartum and is rarely addressed in routine care [32, 33]. Limited time and training for sexual health discussion among midwives are documented barriers [34, 35], suggesting an important area for service development, education and clinical dialogue.

Awareness of CMC services varied considerably. Some women discovered support only after discharge, while others accessed postnatal classes that facilitated peer connection, normalization of emotions and empowerment. This resonates with research emphasizing that information pathways strongly determine service uptake, continuity of care and equitable access [36, 37]. Enhancing communication, especially antenatally, could reduce informational barriers and widen access. The literature further suggests that communication strategies relying solely on information delivery may be insufficient unless accompanied by relationship-building and shared decision-making opportunities - elements that strengthen trust and engagement [37].

While these relational mechanisms appear consistent across settings, the Community Midwifery Care model examined here is embedded within the Italian public health system and reflects specific organizational features, including hospital-community integration and midwife-led home visits in the early postnatal period. These contextual characteristics should be considered when interpreting the transferability of the findings to other healthcare settings.

### Strengths and limitations of this study

A key strength of this research is the rich, narrative-based understanding generated through thematic analysis, capturing not only what women experienced but how they made sense of it. The variety of experiences across breastfeeding, neonatal care, maternal wellbeing and sexuality provides insight into diverse support needs.

However, as a qualitative study conducted within a single Community Midwifery Care service, findings are context-specific and not intended to be statistically generalizable. The characteristics of the sample and care setting may therefore limit transferability to other contexts. Participants self-selected into the study and may have been more likely to report positive experiences of care. In addition, women who did not access or engage with CMC were not included, potentially under-representing unmet needs or negative experiences. Experiences were also captured within an early postnatal timeframe; longitudinal research could explore how perceptions of care evolve over time. Therefore, findings should be interpreted as contextually situated insights into women’s experiences of CMC rather than generalizable outcomes of postnatal home visiting programmes.

### Implications for practice, research and policy

Findings highlight the importance of timely and relational midwifery support that balances clinical guidance with emotional presence. Increasing antenatal awareness of CMC services, strengthening communication pathways, and incorporating routine discussion on sexuality and emotional wellbeing could improve care quality. Postnatal groups emerged as spaces of empowerment and connection; expanding their accessibility may support recovery and reduce isolation. Future research is needed to explore equity of access, partner involvement, and long-term outcomes for mothers and infants.

## Conclusions

Community Midwifery Care supported women across physical, emotional and relational dimensions of early post-partum period. Midwives acted as facilitators of confidence, agents of normalisation and accessible guides in situations of uncertainty. Women valued being listened to, not judged, and supported through practical demonstrations and empathic communication.

Strengthening visibility, continuity and family-oriented support within CMC pathways could enhance maternal wellbeing and contribute to a positive postnatal experience.

## Data Availability

The interview data collected during the current study are available from the corresponding author on reasonable request.
